# Comparing the effects of smartphone-based and face-to-face pulmonary rehabilitation education on caregiver burden and quality of life among the family caregivers of patients with chronic obstructive pulmonary disease: a randomized controlled field trial

**DOI:** 10.1186/s13063-023-07239-7

**Published:** 2023-03-22

**Authors:** Mobina Bahadori, Ramin Sami, Shahla Abolhassani, Vajihe Atashi

**Affiliations:** 1 School of Nursing and Midwifery, Isfahan University of Medical Science, Isfahan, Iran; 2 Department of Internal Medicine, School of Medicine, Isfahan University of Medical Science, Isfahan, Iran; 3 Adult Health Nursing Department, School of Nursing and Midwifery, Nursing and Midwifery Care Research Center, Isfahan University of Medical Science, Isfahan, Iran; 4 Nursing and Midwifery Care Research Center, Adult Health Nursing Department, School of Nursing and Midwifery, Isfahan University of Medical Science, Isfahan, Iran

**Keywords:** Chronic obstructive pulmonary disease, Smartphone-based education, Family caregiver, Caregiver burden, Quality of life

## Abstract

**Background:**

Functional limitation among patients with chronic obstructive pulmonary disorder (COPD) and their dependence on their family caregivers (FCs) can significantly increase caregiver burden (CB) and reduce the quality of life (QOL) among FCs. Education of pulmonary rehabilitation (PR) to FCs is a strategy with potential positive effects on CB. This study was conducted to compare the effects of smartphone-based and face-to-face (FTF) PR education on CB and QOL among the FCs of patients with COPD.

**Methods:**

This randomized controlled field trial was conducted in 2021–2022. Participants were purposefully selected from the PR unit of Khorshid comprehensive respiratory care clinic in Isfahan, Iran, and randomly allocated to a control and an intervention group. Participants in the control group received PR education in twelve 30–60-min FTF sessions held twice weekly in six consecutive weeks. Their counterparts in the intervention group received PR education for 6 weeks through an android application. The Zarit Burden Interview and the 12-item Short Form Health Survey (SF-12) were used for data collection before and immediately after the study intervention. The SPSS software (v. 24.0) was used to analyze the data through the independent-sample *t*, paired-sample *t*, chi-square, and Fisher’s exact tests.

**Results:**

The means of participants’ age was 47.7 ± 13.8 years in the control group and 44.1 ± 14.8 years in the intervention group. Most participants in these groups were female (82.9% vs. 71.4%). The pretest mean scores of CB and QOL were respectively 50.77 ± 10.64 and 27.82 ± 3.9 in the control group and 49.77 ± 7.65 and 26.71 ± 3.5 in the intervention group with no significant between-group difference (*P* > 0.05). At the posttest, these values were respectively 51.57 ± 7.32 and 27.74 ± 3.28 in the control group and 37.31 ± 6.95 and 34.37 ± 2.8 in the intervention group, and between-group differences were significant (P < 0.05). The mean scores of CB and QOL did not significantly change in the control group (*P* > 0.05), but respectively decreased and increased significantly in the intervention group (*P* < 0.05).

**Conclusions:**

Smartphone-based PR education is effective in significantly decreasing CB and improving QOL among the FCs of patients with COPD.

**Trial registration:**

Iranian Registry of Clinical Trials IRCT20161203031200N3

## Background

Chronic obstructive pulmonary disease (COPD) is one of the most prevalent chronic diseases throughout the world. It causes almost irreversible airflow restriction in the airways; is associated with dyspnea, cough, and increased sputum production; and consists of chronic bronchitis and emphysema. The most important underlying causes of COPD are tobacco smoking and air pollution [1]. Currently, 9–10% of the total adult population of the world, i.e., around eighty million people, suffer from COPD [2]. The prevalence of COPD in Iran is also 9–10% [3].

COPD has significant effects on respiration and functional status. COPD-induced dyspnea varies in severity from dyspnea at activity without any significant effect on activity level to complete inability to perform activities due to dyspnea [4]. More than 70% of afflicted patients have problems in daily activities so that those with severe COPD may experience dyspnea and early fatigue even with walking at home [5]. The prevalence of fatigue among patients with COPD is 50–95%. Fatigue significantly restricts the ability to perform physical activity [6].

COPD chronicity, continuous reduction of functionality, and long-term courses of COPD management require the active involvement of family members in the process of COPD management [7]. Family caregivers (FCs) need to monitor COPD symptoms, supervise the accurate use of medications, assess the symptoms of COPD exacerbation, and arrange periodical medical visits [8]. However, the progressive dependence of COPD-afflicted patients on their FCs can negatively affect the personal life of FCs, undermine their physical and mental health, and increase their caregiver burden (CB) [9]. CB is defined as physical, mental, and social reactions of FCs to the imbalance between caregiving requirements and their own personal needs and tasks [10]. A study reported that 21% of the FCs of patients with COPD suffered from social limitations, 38.9% of them had reduced their work hours, and 50% of them suffered from alterations in their daily activities [8]. Moreover, increasing physical, mental, and financial pressures due to COPD exacerbation make FCs ignore their health and self-care and reduce their QOL. A study indicated that the FCs of patients with COPD had heavy CB and low QOL [11].

Providing education about pulmonary rehabilitation (PR) to the FCs of patients with COPD is a strategy with potential positive effects on their QOL. PR is a multidisciplinary approach to improve functional status and independence at activities and is the main component of COPD management. It has significant positive effects on COPD symptoms and quality of life (QOL) [12]. A study reported that PR significantly improved the level of daily activities and QOL among patients with COPD [13]. Another study on patients with COPD showed that PR significantly reduced the symptoms of fatigue and dyspnea [6].

There are different methods to provide PR education to the FCs of patients with COPD, including face-to-face (FTF) education, web-based education, and smartphone-based education [14]. FTF education is the standard method for education [15]. However, this method cannot completely fulfill the needs of the FCs of patients with chronic diseases [16]. Moreover, many FCs cannot attend FTF education sessions due to long distances and time limitations or may easily forget educational materials due to the lack of continuity in education. These problems in turn reduce their participation in educational programs [15].

One of the modern methods for PR is smartphone applications [17]. As a support system for FCs, these applications can improve their caregiving knowledge and empower them for patient support [18]. Moreover, these applications do not have the shortcomings of FTF PR education such as time wastage, forgetfulness of educational materials, and necessity to travel long distances to receive educations [19]. Another study found the effectiveness of a smartphone-based educational intervention in significantly reducing CB among the lay caregivers of patients with chronic diseases [15].

Despite the known positive effects of education on caregiving knowledge among FCs of chronically ill patients, some studies reported that the FCs of patients with COPD did not have adequate PR knowledge and needed further information for quality caregiving to their patients [20]. Most previous studies into the effects of education on family caregivers were not smartphone-based and were conducted on patients with chronic diseases other than COPD [21]. Besides, although some studies assessed the effects of smartphone-based interventions on QOL among patients with COPD [22], there is limited information on the effects of such interventions on the needs and QOL of their FCs [23]. These gaps highlight the necessity of further studies in this area. Therefore, the present study was conducted to compare the effects of smartphone-based and FTF PR education on CB and QOL among the FCs of patients with COPD.

## Methods

### Design and participants

This randomized controlled field trial was conducted in 2021–2022. The study setting was Khorshid comprehensive respiratory care clinic in Isfahan, Iran, and the study population consisted of all FCs of patients with COPD who were referred to the PR unit of this clinic. Sampling was purposefully performed, and eligibility criteria were age of 18–65 years; having a caregiver live with the patient; basic literacy skills; access to the Internet and smartphone; ability to use a smartphone; no hearing, visual, or cognitive problems; agreement for participation in the study; and history of caregiving to a patient with COPD for at least 6 months. The exclusion criteria were reluctance to stay in the study, more than two absences from the intervention sessions, having other chronic diseases along with COPD in patients, inability to access the Internet or smartphone, and death of FC or patient during the study.

**Fig. 1:**
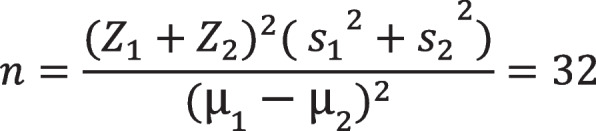
Sample size calculation

Based on the results of a previous study [24] and with a confidence level of 0.95, a power of 0.8, and an effect size of 0.9, the sample size was calculated to be 32 per group (Fig. 1). However, considering a 20% probable withdrawal rate, the sample size was increased to 38 per group. The Minimization software was used to randomly allocate participants to a control and an intervention group based on their age, gender, and educational level.

$$n=\frac{({{Z}_{1}+{Z}_{2})}^{2}{{( {s}_{1}}^{2}+{s}_{2}}^{2})}{{({\mu }_{1}-{\mu }_{2})}^{2}}=32$$

### Instruments

The instruments were a demographic questionnaire, the Zarit Burden Interview, and the 12-item Short Form Health Survey (SF-12). The demographic questionnaire had items on participants’ demographic characteristics such as age, gender, occupation, place of residence, marital status, kinship with patient, duration of caregiving, and affliction by chronic diseases.

Zarit Burden Interview is a self-report instrument with 22 items in five subscales, namely burden in the relationship, emotional well-being, social and family life, finances, and loss of control over one’s life. Items are scored on a 5-point scale from zero (“never”) to 4 (“almost always”), and the possible total score of the instrument is 0–88 with higher scores showing heavier CB. The total score of this instrument is categorized and interpreted as follows: scores 0–20: light CB; scores 21–40: mild to moderate CB; scores 41–60: moderate to heavy CB; and scores 61–88: heavy CB. Previous studies confirmed the acceptable validity and reliability of the Persian version of this instrument [25, 26].

SF-12 was used to assess participants’ QOL. This survey has twelve items in two main dimensions, namely physical health and mental health. The physical health dimension has items on limitations in physical role functioning, physical health, general health perception, and bodily pain. The items of the mental health dimension are limitation in emotional role functioning, vitality, mental health, and social functioning. Items are scored on 1–2, 1–3, 1–5, or 1–6 scales, and the possible total score of the survey is 12–48, which is interpreted as follows: 12–24: poor QOL; 25–36: moderate QOL; and 37–48: high QOL. Previous studies confirmed that the Persian version of this survey has acceptable validity and reliability [27, 28].

### Intervention

The study intervention was a PR educational program developed based on the existing literature [4, 29, 30] and based on the educational needs of FCs. Four experts in PR (i.e., a lung specialist, a nurse, a physical medicine specialist, and a psychiatrist) evaluated and confirmed the validity of the program. Educational materials were about self-care, dyspnea management, oxygen therapy, use of inhaled medications, relaxation, and physical activities which improve respiratory functioning. A PR nurse provided educational materials to participants in the control group through the FTF method in twelve 30–60-min sessions held twice weekly in six consecutive weeks. Each week during the intervention, two telephone contacts were made with participants in the control group to remind them to participate in the intervention sessions.

In the intervention group, participants received educational materials through an android application which could be used both offline and online. The application had two main panels, namely an administrator panel and a user panel. The administrator panel included modification of program content and parts, adding or deleting users, answering the questions, monitoring the results of patients’ diagnostic tests, and monitoring the amount of using educational materials by each user. The user panel included video and written materials on PR, instruction to use different inhaled medications, physical exercises to improve respiratory functioning, relaxation, other caregiving-related tips, asking questions, sending the results of diagnostic tests, daily documentation of vital signs, and daily reminder messages. Participants needed to complete a knowledge assessment test before accessing PR educational materials. The application was installed on participants’ smartphones, where they could use it for six consecutive weeks. Before using the application, participants were provided with instructions about how to use it in a FTF session. Twice weekly telephone contacts were also made with participants in the intervention group to assess their needs and status. Participants in both groups completed the study instruments before and immediately after the study intervention.

### Data analysis

Data were analyzed using the SPSS software (v. 24.0). Between-group comparisons respecting participants’ demographic characteristics were made using the independent-sample *t*, chi-square, and Fisher’s exact tests. Between- and within-group comparisons respecting the mean scores of CB and QOL were also made using the independent-sample *t* and the paired-sample *t* tests, respectively. The level of significance was set at less than 0.05.

## Results

**Fig. 2:**
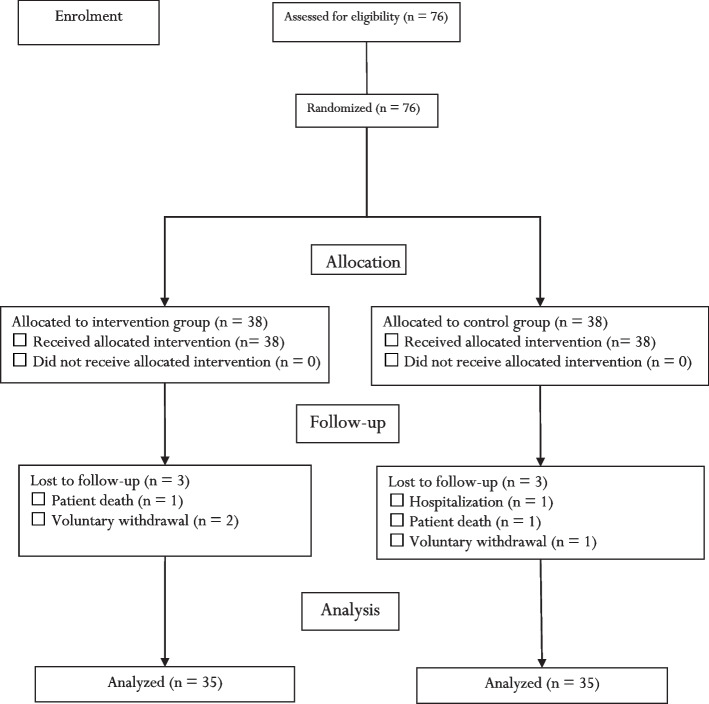
The flow diagram of the study

Initially, 76 eligible FCs of patients with COPD were recruited for the study. Three participants from the intervention group were excluded due to reluctance to use the application (*n* = 2) and patient death (*n* = 1), and three participants from the control group were excluded due to patient death (*n* = 1), hospitalization (*n* = 1), and reluctance to stay in the study (*n* = 1). Finally, 35 participants in each group (i.e., seventy in total) were included in the final analysis (Fig. 2).

The means of participants’ age and duration of caregiving were 47.7 ± 13.8 and 4.2 ± 4.8 years in the control group and 44.1 ± 14.8 and 4.4 ± 5.6 years in the intervention group. Most participants in these groups were female (82.9% vs. 71.4%) and patients’ spouse (51.42% vs. 62.85%) and did not report a history of affliction by chronic diseases (62.9% vs. 54.3%). Groups did not significantly differ from each other respecting participants’ demographic characteristics (*P* > 0.05) (Table 1).

**Table 1:** Between-group comparisons concerning participants’ characteristics

Groups; characteristics		Intervention, mean ± SD or *N* (%)	Control, mean ± SD or *N* (%)	Test value	*P* value
Age (years)		44.14 ± 14.08	47.71 ± 13.84	1.07	0.289
Duration of caregiving (years)		4.4 ± 5.6 Median (IQR): 3 (3)	4.2 ± 4.8 Median (IQR): 3 (4)	0.160	0.599
Gender	Female	25 (71.4)	29 (82.9)	1.296	0.255
	Male	10 (28.6)	6 (17.1)		
Affliction by chronic disease	Yes	19 (54.3)	22 (62.9)	0.530	0.467
	No	16 (45.7)	13 (37.1)		
Occupation	Self-employed	10 (28.6)	16 (45.71)	4.88	0.181
	Employee	13 (37.1)	9 (25.7)		
	Unemployed	–	2 (5.71)		
	Housewife	10 (28.6)	5 (14.3)		
	Retired	2 (5.7)	3 (8.6)		
Kinship with patient	Son	5 (14.28)	6 (17.14)	0.530	0.549
	Daughter	7 (20)	10 (28.57)		
	Daughter-in-law	1 (2.58)	1 (2.58)		
	Spouse	22 (62.85)	18 (51.42)		
Educational level	Primary	5 (14.3)	6 (17.1)	0.107	0.937
	Secondary	8 (22.9)	6 (17.1)		
	Diploma	12 (34.3)	13 (37.1)		
	Bachelor’s	8 (22.9)	9 (25.7)		
	Master’s and higher	2 (5.7)	1 (2.9)		

The pretest mean score of CB was 50.77 ± 10.64 in the control group and 49.77 ± 7.65 in the intervention group with no significant between-group difference (*P* = 0.653). After the intervention, the mean score of CB was 51.57 ± 7.32 in the control group and 37.31 ± 6.95 in the intervention group. The posttest mean score of CB in the intervention group was significantly less than the control group (*P* < 0.001) (Table 2). Within-group comparisons also revealed that the mean score of CB did not significantly change in the control group (*P* = 0.573) but significantly decreased in the intervention group (*P* < 0.001).

**Table 2:** Between- and within-group comparisons respecting the mean scores of caregiver burden and quality of life

Outcomes	Time; group	Before	After	*P* value
Caregiver burden	Control	50.77 ± 10.64	51.57 ± 7.32	0.573
	Intervention	7.65 $\pm$ 49.77	6.95 $\pm$ 37.31	0.001
	*P* value	0.653	0.001	
Total quality of life	Control	3.9 $\pm$ 27.82	3.28 $\pm$ 27.74	0.829
	Intervention	3.5 $\pm$ 26.71	2.8 $\pm$ 34.37	0.001
	*P* value	0.604	0.001	
Physical quality of life	Control	12.71 $\pm$ 2.34	2.03 $\pm$ 12.7	0.98
	Intervention	2.23 $\pm$ 12.37	1.57 $\pm$ 14.42	0.001
	*P* value	0.543	0.001	
Mental quality of life	Control	2.16 $\pm$ 15.11	1.99 $\pm$ 15.02	0.774
	Intervention	2.22 $\pm$ 14.43	1.86 $\pm$ 19.94	0.001
	*P* value	0.146	0.001	

The pretest mean score of QOL in the control and the intervention groups was respectively 27.82 ± 3.9 and 26.71 ± 3.5 and the between-group difference was not significant (*P* = 0.604). The posttest mean score of QOL in these groups was respectively 27.74 ± 3.28 and 34.37 ± 2.8, and the between-group difference was statistically significant (*P* < 0.001). Within-group comparisons indicated no significant change in the mean score of QOL in the control group (*P* = 0.829) and significant increase in this mean score in the intervention group (*P* < 0.001) (Table 2).

## Discussion

This study compared the effects of smartphone-based and FTF PR education on CB and QOL among the FCs of patients with COPD. Findings indicated that FTF PR education had no significant effects on CB and QOL, while smartphone-based PR education significantly reduced CB and improved QOL among the FCs of patients with COPD.

Our findings showed that most FCs of patients with COPD were female. Findings also indicated that most FCs of patients with COPD were their spouses. In line with this finding, a study revealed that 58% of the FCs of patients with COPD were their spouses. The QOL of patients with chronic diseases and that of their caregiving spouses are affected by each other, and hence, spouses who are involved in caregiving may be more vulnerable [31].

Our findings also revealed that there was no significant between-group difference respecting the pretest mean score of CB while the posttest mean score of CB in the intervention group was significantly less than the control group. This finding denotes the insignificant effects of FTF PR education and the significant positive effects of smartphone-based PR education on CB among the FCs of patients with COPD. In agreement with this finding, a study found that smartphone-based education was effective in significantly reducing fatigue, stress, and CB among the FCs of individuals with dementia [32]. Another study showed that smartphone-based education significantly reduced depression symptoms and CB among the FCs of patients with heart failure [33]. The effectiveness of smartphone-based educational interventions in reducing CB may be due to the fact that these interventions fulfill the educational needs of FCs, improve their disease-related knowledge, empower them for caregiving, improve their ability to manage caregiving challenges, and thereby reduce their stress [18]. Compared with FTF education, tele-education has many positive effects such as lower costs, no need for traveling to receive education, no time wastage, easy access to educational materials at any time and place, and lower CB [32]. A study reported that educations which are based on the needs of FCs and are provided using the most appropriate methods can reduce caregiving challenges among FCs and reduce their CB [33].

Contrary to our findings, a study showed that web-based education for 3 months had no significant effects on CB among the FCs of patients with Alzheimer’s disease [34]. Another study also reported the insignificant effects of web-based education on CB and QOL among the FCs of patients with dementia [35]. This contradiction can be due to the difference among studies respecting the underlying conditions of patients. Although both dementia and Alzheimer’s disease are chronic conditions, they considerably differ from other chronic diseases such as COPD with respect to caregiving challenges. Dementia and Alzheimer’s disease are brain disorders which affect cognition, memory, and problem solving, while COPD is a physical disease with progressive irreversible restriction of airflow in the airways. Evidence shows that although caregiving to patients with dementia and patients with COPD is difficult and stressful, the FCs of patients with dementia and Alzheimer’s disease spend more time on caregiving, have lower ability to cope with their conditions, and experience more serious physical, emotional, and financial problems [36]. Another explanation for the contradiction among studies may be their difference in respecting their intervention. Our intervention was a smartphone-based PR education while the intervention of those two studies was web-based education [34, 35]. Access to educational materials in smartphone-based education is easier than web-based education because smartphone-based education can be provided offline, while web-based education needs access to high-speed internet [18].

We also found that although there was no significant between-group difference respecting the pretest mean score of QOL, the posttest mean score of QOL in the intervention group was significantly more than the control group. In agreement with this finding, one study showed that smartphone-based education significantly improved QOL among the FCs of patients with Alzheimer’s disease [37]. Smartphone-based educational programs for FCs are usually developed based on their educational needs, help healthcare providers support them, enable them to more easily access caregiving-related information [21], empower them for caregiving, and enhance their satisfaction with and motivation for caregiving [15]. These advantages of smartphone-based educational programs can bring positive behavioral changes and thereby improve QOL among FCs[37].

In contradiction to our findings, a study showed the insignificant effects of a smartphone-based breast cancer support program on QOL among women with breast cancer who received chemotherapy [38]. This contradiction is attributable to the difference between the studies in terms of the underlying disease of patients which was COPD in the present study and breast cancer in that study. Breast cancer mostly affects women [39], while COPD mostly affects men [2]. Such gender differences can affect the effects of chronic diseases on the different aspects of life [1]. Moreover, most FCs of women with breast cancer are their husbands, and the negative effects of caregiving may be more severe among men [39]. Another explanation for this contradiction between the studies is that our study was conducted on patients’ FCs, while participants in that study were patients with breast cancer. Although FCs are considerably affected by problems such as psychological distress and are considered as latent patients, patients with chronic diseases experience a wide range of physical and psychosocial problems due to their underlying conditions [9].

We also found that FTF PR education had no significant effects on CB and QOL among the FCs of patients with COPD. In agreement with this finding, a study into the effects of caregiving education revealed that routine family education had no significant effects on CB among the FCs of patients with stroke [40]. The insignificant effects of routine FTF education on CB among FCs in the present study may be due to the fact that educations were provided only twice weekly, and participants might have forgotten educational materials. On the other hand, participants in the intervention group had free access to smartphone-based educational materials and could freely study and review them.

### Study limitations

One of the study limitations was the paucity of patients with COPD in the study setting due to the coronavirus disease 2019 pandemic which prolonged the process of sampling. Moreover, the pandemic conditions had required the authorities of the study setting to reduce care services for patients with COPD in order to provide care to patients with coronavirus disease 2019. Besides, among the eligibility criteria in this study were access to the Internet and the ability to use a smartphone, while some FCs of patients with COPD in the study setting did not have access to the Internet or a smartphone.

## Conclusions

Smartphone-based PR education is effective in significantly reducing CB and improving QOL among the FCs of patients with COPD. This intervention is easily accessible anywhere and at any time, improves access to healthcare providers and educational materials, and can improve motivation for caregiving through interaction with healthcare providers. Given the heavy costs of FTF healthcare services, paucity of professional respiratory care centers, and non-fulfillment of most educational needs of patients and their FCs during FTF education, smartphone-based PR education can be used for more effective need fulfillment and problem management among patients and their FCs.

## Data Availability

The datasets analyzed during the current study are available from the corresponding author upon reasonable request.

## Abbreviations

COPD: Chronic obstructive pulmonary disorder
FCs: Family caregivers
CB: Caregiver burden
QOL: Quality of life
